# Systemic removal of host restriction factors enables rapid, scalable virus production for cell culture-based vaccine development

**DOI:** 10.3389/fbioe.2026.1877497

**Published:** 2026-08-21

**Authors:** Zhengmei Xu, Jaemyeong Jeon, Jinsoo Oh, Youngki Shin, Se-Ho Park

**Affiliations:** Division of Life Sciences, College of Life Sciences and Biotechnology, Korea University, Seoul, Republic of Korea

**Keywords:** cell culture-based vaccines, CRISPR-Cas9, high-yield virus production, host restriction factors, multi-gene knockout, RNase L, PKR, JAK1

## Abstract

The efficiency of cell culture-based vaccine production is fundamentally constrained by the antiviral defenses of host cells, creating a major bottleneck for rapid and large-scale vaccine manufacturing. Key antiviral proteins-such as RNase L, PKR, and JAK1 act as intrinsic brakes on viral replication, limiting efficient propagation of many clinically relevant viruses. To overcome this challenge, we generated HEK293T, Vero, and MDCK cell lines with targeted knockouts of multiple antiviral genes. Notably, these engineered cells maintained normal growth and viability while supporting markedly increased viral yields. Multi-gene deletions enhance the replication of both enveloped viruses, including influenza A virus, pseudotyped lentivirus, and porcine epidemic diarrhea virus (PEDV), and non-enveloped viruses such as coxsackievirus. The magnitude of enhancement scaled proportionally with the number of genes disrupted. By systematically removing host restriction factors, this platform provides a versatile and powerful strategy for accelerating viral propagation, offering a strong foundation for more efficient development and large-scale production of cell culture-based anti-viral vaccines.

## 1 Introduction

Vaccines are one of the most effective tools for preventing infectious diseases (Gallagher and Lipsitch, 2019). However, the efficiency of vaccine manufacturing remains a critical bottleneck in global health preparedness (Peletta et al., 2023; Williams et al., 2023). Cell culture-based systems, which form the foundation of most modern vaccine production, face substantial challenges in achieving high viral yields (Zinnecker et al., 2024). Commonly used cell lines, such as Vero and MDCK, often have limited viral productivity, primarily due to the host cells’ potent intrinsic antiviral defense mechanisms (Duggal and Emerman, 2012). These defense mechanisms, including interferon (IFN) signaling and restriction factor expression, actively suppress viral replication (Randall and Goodbourn, 2008). Advances in gene editing, particularly CRISPR-Cas9, provide a powerful means to overcome this barrier by engineering host cells to be more permissive to viral replication (Hu et al., 2024). Promising results have been obtained from the first proof-of-concept study disrupting BST2, which tethers the host cells and viral membrane thus limiting virus release from the host cells (Yi et al., 2019). However, because many viruses possess viral elements that counteract specific host restriction factors, it is difficult to restrict a wide range of viruses with a single restriction factor. Therefore, host cells possess a variety of restriction factors throughout the viral life cycle, effectively suppressing viral replication.

While proof-of-concept studies, including *BST2* knockout to enhance the release of enveloped viruses, have shown promise, the redundancy of the host antiviral network inherently limits such single-target approaches (Yi et al., 2019). In fact, BST2 can only act on a subset of enveloped viruses. Non-enveloped viruses are not restricted by BST2 (Le Tortorec et al., 2011), and certain enveloped viruses, such as human immunodeficiency virus 1 (HIV-1), degrade host BST2 with viral Vpu protein (Van Damme et al., 2008). To systematically dismantle this redundant defense network, we targeted key nodes of the innate immune system: *PKR* (García et al., 2006), *RNase L* (Silverman, 2007), and *JAK1* genes in addition to *BST2*. Protein kinase R (PKR) is a host enzyme activated by double-stranded RNA (dsRNA) during viral infection. Activated PKR phosphorylates the translation factor eIF2α, which blocks viral replication by halting protein synthesis. It can also induce host cell apoptosis and activate NF-κB signaling to enhance antiviral gene expression (Sadler et al., 2009; Martínez et al., 2025). RNase L is a host enzyme activated by 2′,5′-oligoadenylate (2–5A), which is produced by oligoadenylate synthetase (OAS) in response to viral double-stranded RNA (dsRNA). RNase L degrades viral and host RNA, halting viral replication and producing small RNA fragments that amplify interferon signaling (Silverman, 2007). Janus Kinase 1 (JAK1) is a key host kinase in the interferon signaling pathway initiated by viral infection. When interferon binds to its receptor, JAK1 along with TYK2 phosphorylates STAT1/STAT2, which forms the ISGF3 complex, establishing an antiviral state within the cell by inducing the expression of interferon-stimulated genes (*ISGs*) (Au-Yeung et al., 2013; Hu et al., 2021). BST2 (also known as tetherin or CD317) is an interferon-induced antiviral protein that restricts the release of many enveloped viruses from infected cells. It acts by physically “tethering” budding virions to the cell membrane, preventing their release and subsequent infection of new cells (Evans et al., 2010).

We generated knockout cell lines for these genes in three cell models with industrial applications: HEK293T, a human kidney cell line used to produce pseudotyped lentivirus for gene transfer; Vero, a monkey kidney cell line used to produce various antiviral vaccines; and MDCK, a canine kidney cell line which is the major cell line used to produce anti-influenza vaccines. In all these cell lines, multiple antiviral gene knockouts significantly increased virus production for a wide range of viruses, including both enveloped and non-enveloped viruses. This cross-species systematic multi-gene knockout virus production platform (collectively, the platform) will provide robust productivity to produce antiviral vaccines and therapeutic vectors (Liu and Moss, 2016; Jones et al., 2021).

## 2 Materials and methods

### 2.1 Cell lines and gene knockout

MDCK.1 (ATCC-CRL-2935), Vero (ATCC-CCL-81), HEK293T (ATCC-CRL-3216) cells, and their respective multi gene deficient cell lines-HEK293T (B, BR, BRJ), Vero (B, BP, BPR, BPRJ), MDCK (B, BR, BRJ) were cultured in Dulbecco’s modified Eagle’s medium (DMEM, Gibco) supplemented with 10% FBS (Hyclone), 2 mML-glutamine (Gibco), 1% penicillin-streptomycin (Gibco), 10 μg/mL Gentamycin (Gibco), and 50 mMb-mercaptoethanol (Gibco) at 37 °C with 5% CO_2_. Generation of BST2-deficient cell lines HEK293T-B, Vero-B, and MDCK-B has been described previously (Yi et al., 2017).

Gene knockouts were generated using the pSpCas9(BB)-2A-GFP (PX458) backbone which produces Cas9 enzyme and sgRNA simultaneously. For each target gene, sgRNA expression plasmids (pX458-sgRNA (Target gene)-Cas9(BB)-T2A-GFP) were constructed and transfected into cells with Turbofect (Thermo Fisher Scientific). Candidate sgRNA target sites were designed using CHOPCHOP (https://chopchop.cbu.uib.no/) based on predicted on-target activity and off-target potential. GFP-positive cells were enriched by fluorescence-activated cell sorting (BD FACSAria II) and seeded into 96-well plates for single-cell cloning. PCR products obtained from the genomic DNA of each clone were heated at 95 °C for 5 min and then treated with T7 endonuclease I (T7E1) at 37 °C for 30 min. All mismatched pairs resulting from the insertion or deletion of the target gene are cleaved by endonucleases (PCR primer sequences are included in the supplementary table). Clones showing cleavage patterns were further validated by Sanger sequencing (Bioneer), and homozygous knockout lines were confirmed.

Cells used for growth analysis were passaged approximately 20 times before the assay. The cells were seeded into 96-well plates, and cell viability was measured daily using the CCK-8 assay. The optical density (OD) was recorded to determine the growth rate of each cell line.

### 2.2 Virus production and viral titer determination

Seasonal influenza H1N1 (A/Brisbane/59/2007) and H3N2 (A/Brisbane/10/2007) viruses were obtained from the Korea Centers for Disease Control and Prevention (KCDC), and Yamagata (B/Wiscosin/1/2010) was obtained from the National Biobank of Korea (NBK). Coxsackievirus B4 was obtained from the Korea Disease Control and Prevention Agency (KDCA). For infection by the influenza virus, we followed the previous protocol (Yi et al., 2017). Porcine epidemic diarrhea virus (strain CV777), provided by the Korea Veterinary Culture Collection (KVCC), was used to infect cells. Varicella zoster virus (VZV-Oka) was obtained from the National Institute of Food and Drug Safety Evaluation (NIFDS) of South Korea. In brief, cells were seeded 20–24 h before infection. When cells reached 90% confluence, they were washed with PBS. Next, cells were inoculated with 0.001–0.1 MOI of virus containing 0.3% BSA and 1–2 μg/mL of TPCK-trypsin (Sigma) in MEM medium for 1 h. Finally, the virus inoculum was removed and replaced with a fresh medium containing 0.3% BSA and TPCK-trypsin for 48 h at 37 °C under 5% CO_2_. Following incubation, the culture supernatant, containing progeny viruses, was collected, centrifuged to remove cellular debris, and stored at −80 °C until analysis.

Vero cells were seeded in 24-well plates (or specified culture vessel) and allowed to adhere for 20–24 h. At the time of infection, when cells had reached approximately 90% confluency, the culture medium was aspirated, and the cell monolayer was gently rinsed with PBS to remove residual serum. Infection was carried out by incubating the cells with Coxsackievirus B4 at the indicated dose (1.25–2.5 μL of viral stock) in serum-free DMEM. The virus-cell mixture was incubated for 72 h at 37 °C under 5% CO_2_. Following incubation, the culture supernatant, containing progeny viruses, was collected, centrifuged to remove cellular debris, and stored at −80 °C until analysis.

Cytopathic effects (CPE) induced by virus infection were monitored by light microscopy. Infected and mock-infected Vero WT cells were observed daily for CPE using an inverted phase-contrast microscope. At 48–72 h post-infection, representative images were captured at identical magnifications for comparison. For each well, the extent of CPE was estimated by determining the percentage of the cell monolayer exhibiting characteristic cytopathic changes, including cell rounding, detachment, and loss of monolayer integrity. CPE (%) was calculated as the proportion of affected cells relative to the total cell population in each well. The mean CPE percentage ± SEM was calculated from four wells per condition.

Lentiviral particles were produced using a three-plasmid packaging system. The plasmids included pCDH-VSV G as the envelope plasmid, psPAX2 as the packaging plasmid, and either pCDH-copGFP or pCDH-CD19-CAR-mCherry as the transfer plasmid. Viral supernatants were harvested 48 h post-transfection and stored at −80 °C.

Viral titers (TU/mL) were determined by infecting HEK293T WT cells with serial dilutions of the harvested supernatants. Following infection, the proportion of reporter gene–positive cells (copGFP^+^ or Protein L^+^) was quantified by flow cytometry, and the viral titers were calculated as follows: TU/mL = (Number of seeded cells × Percentage of positive cells × Dilution factor)/Volume of virus used (mL).

Vero cells were allowed to adhere in 6-well plates for 20 h before being infected with VZV (4 × 10^3^PFU/well in 2% DMEM). Infected cells were subsequently passaged once a week, and the cells were monitored for the VZV glycoprotein E surface expression by flow cytometry at each passage.

### 2.3 Replicon transfection

Coxsackievirus CVB3 replicons were amplified in *Escherichia coli* and purified using a plasmid purification kit (Bioneer) according to the manufacturer’s instructions. Purified DNA was quantified by spectrophotometry and stored at −20 °C until use.

Cells were seeded 20–24 h before transfection and allowed to reach approximately 70%–80% confluence at the time of transfection. Plasmid DNA was transfected into cells using a lipid-based transfection reagent (Invitrogen Lipofectamine™ 2000) following the manufacturer’s protocol. Briefly, plasmid DNA and transfection reagent were diluted separately in Opti-MEM (Gibco), mixed gently, and incubated at room temperature for 10–20 min to allow complex formation. The DNA–lipid complexes were then added dropwise to the cells in serum-free medium.

After 48 h of incubation at 37 °C under 5% CO_2_, the culture supernatant, containing progeny virus replicon, was collected, centrifuged to remove cellular debris, and stored at −80 °C until analysis.

### 2.4 Establishment of suspension cell lines and virus production

MDCK and Vero cells were adapted to serum-free suspension culture in 125-mL Erlenmeyer shake flasks (Corning-431143) containing 30 mL of medium, and the cultures were agitated at 110 rpm on Lab companion SK-300 orbital shaker (Jeiotech, Daejeon, Korea). MDCK cells were maintained in serum-free CD MDCK 244 medium (KCell Biosciences), while Vero cells were cultured in serum-free DMEM/F12 medium (Gibco-12500062). Adaptation was initiated at 5 × 10^6^ cells and achieved through serial passaging, with the removal of adherent cells, until stable, uniformly growing suspension clones were obtained. For Vero cells adaptation, all cells harvested in the early stage of adaptation were re-inoculated into fresh medium due to poor cell growth, until the total harvested cell number exceeded 2 × 10^6^ cells. 2 × 10^6^ cells were then subcultured into each new culture thereafter.

Following infection with H1N1 influenza virus (A/Brisbane/59/2007) or porcine epidemic diarrhea virus (PEDV, CV777), suspension cells were cultured in serum-free medium under constant agitation at 110 rpm. Supernatants were collected at intervals between 48 and 92-h post-infection, clarified by centrifugation, and analyzed by Western blotting.

### 2.5 Western blotting

Cells were lysed with RIPA Cell Lysis buffer (GenDEPOT) supplemented with Xpert Protease Inhibitor Cocktail Solution (GenDEPOT). Then 10–30 μg of extracts or virus sup were electrophoresed onto a 12% gradient gel (Bio-Rad), transferred to a polyvinylidene difluoride (PVDF) membrane, and incubated with primary antibodies (dilutions 1:1000) in Tris-buffered saline with 5% BSA and 0.1% Tween 20 (TBST). For Western blot analysis, samples were prepared under reducing conditions unless otherwise specified. The membranes were incubated with primary antibodies against STAT1 (Cell Signaling, Cat#9172P), GAPDH (Santa Cruz Biotechnology, Cat#E2419), phospho-STAT1 (Cell Signaling, Cat#9171P), Influenza H1N1 HA (Sinobiological, Cat#11052-MM06), Influenza H3N2 HA (Sinobiological, Cat#11056-MM03), PEDV N (MyBioSource, Cat#MBS560870), and VZV glycoprotein E (Santa Cruz Biotechnology, Cat#K2522), followed by HRP-conjugated secondary antibodies. Protein bands were visualized using enhanced chemiluminescence. Blot images were obtained using the LAS4000 mini (GE Healthcare). Band intensities were analyzed with the ImageJ program. Levels of target proteins were normalized against the level of GAPDH as a loading control.

### 2.6 Statistical analysis

GraphPad Prism Version 6.0 (GraphPad Software) was used to perform statistical analysis. Differences in data were compared using an unpaired two-tailed t-test or a two-way ANOVA with Sidak’s multiple comparison test. Unless otherwise stated, all experiments were performed using three independent biological replicates. Data are expressed as means ± standard errors of the mean (SEM). Differences were considered significant if p < 0.05.

## 3 Results

### 3.1 Systematic elimination of host antiviral defense genes

**FIGURE 1:**
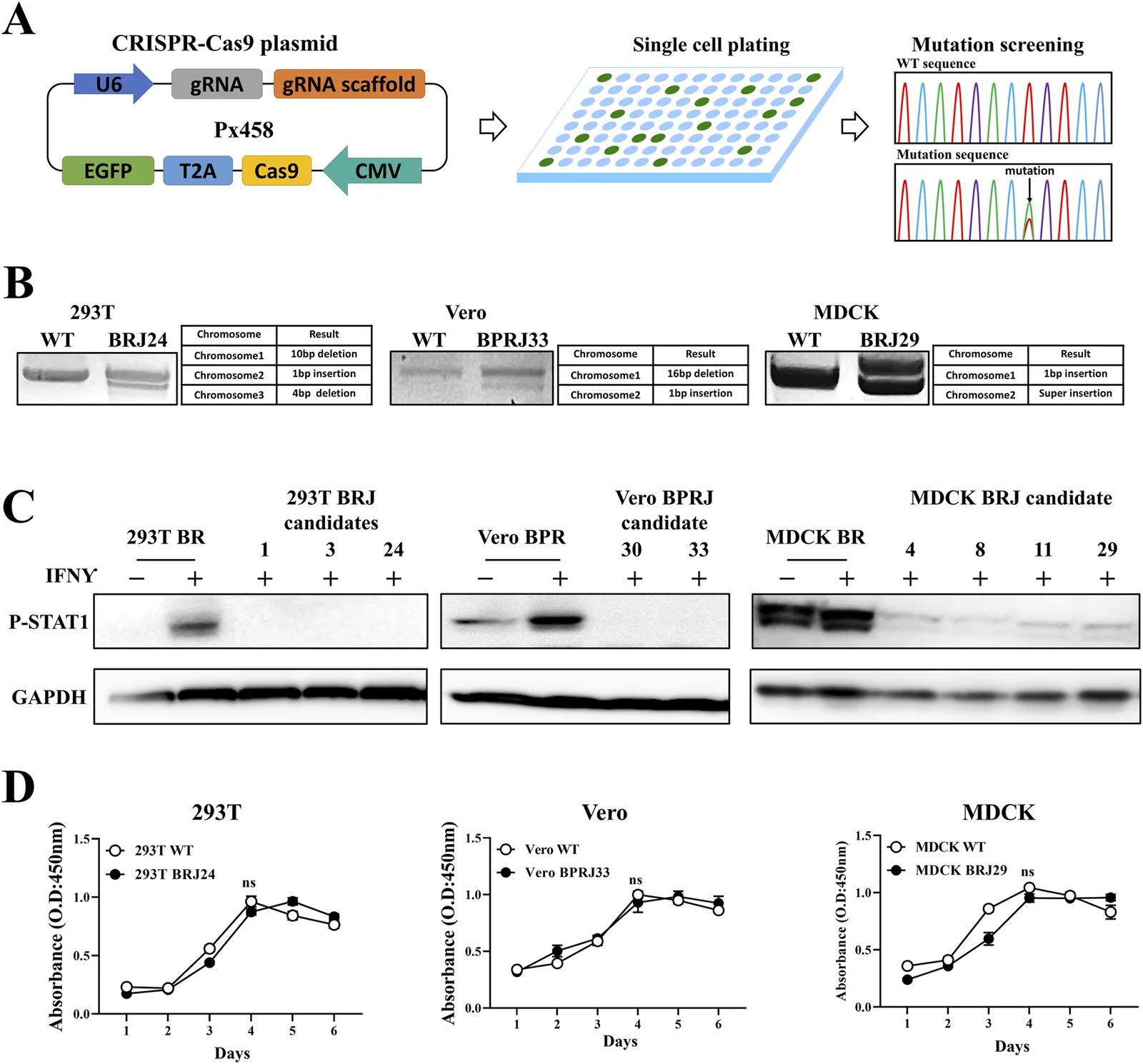
Generation and functional characterization of antiviral gene knockout cell models. **(A)** Workflow for generating single-clone knockout cell lines. Guide RNA for the target gene was cloned into pSpCas9(BB)-2A-GFP(PX458) vector. After transfection with the gene-targeting vector, GFP-positive cells were isolated via FACS and single-cell cultured. **(B)** Effective mutagenesis was confirmed by T7E1 analysis, and the presence of a homozygous frameshift mutation was verified by Sanger sequencing. **(C)** JAK1 knockout abolished IFNγ-induced STAT1 phosphorylation. **(D)** Normal cell growth of mutant cell lines. CCK8 assay optical density (O.D.) values indicate normal proliferation capacity of knockout cells compared to wild-type cells. ns, not significant.

We employed the CRISPR-Cas9 system to generate targeted knockouts of key antiviral genes in HEK293T, Vero, and MDCK cells. Specific sgRNAs were cloned into the pX458 vector, and monoclonal cell lines were subsequently isolated through sorting GFP-positive cells and limited dilution (Figure 1A). All gene-targeting sequences used in this study, including target sequences, are provided in the Supplementary Table). In this study, we additionally knocked out *PKR, RNase L* and *JAK1* genes in the previously established *BST2* knockout cell lines (Yi et al., 2017). In each cell line, the following knockout combinations were established: *BST2* (B), *BST2* and *PKR* (BP), *BST2* and *RNase L* (BR), *BST2*, *PKR*, and *RNase L* (BPR), and *BST2*, *PKR*, *RNase L*, and *JAK1* (BPRJ), with nomenclature reflecting the targeted genes in each cell line. Targeted mutations were verified by T7 endonuclease 1 (T7E1) digestion (Figure 1B), and Sanger sequencing of representative clones confirmed homozygous frameshift mutations (Supplementary Table S2). To validate the functional disruption of target genes, we examined protein expression in 293T cells after knocking out *RNase L*. Western blot analysis confirmed the disappearance of RNase L protein upon interferon γ (IFN-γ) stimulation in *RNase L*-deficient cells (Supplementary Figure S1A). To verify the function of JAK1, we assessed the phosphorylation pattern of Signal Transducer and Activator of Transcription 1 (STAT1), a key downstream signaling molecule of JAK1. Western blot analysis revealed significantly reduced STAT1 phosphorylation (p-STAT1) in *JAK1*-deficient cells following IFN stimulation, confirming inhibition of the JAK-STAT signaling pathway (Figure 1C). Despite the targeted mutations, cell proliferation assays revealed that these gene knockouts did not impair normal cell growth or viability (Figure 1D).

### 3.2 Potent activation of enveloped virus replication through the stepwise elimination of antiviral genes

**FIGURE 2:**
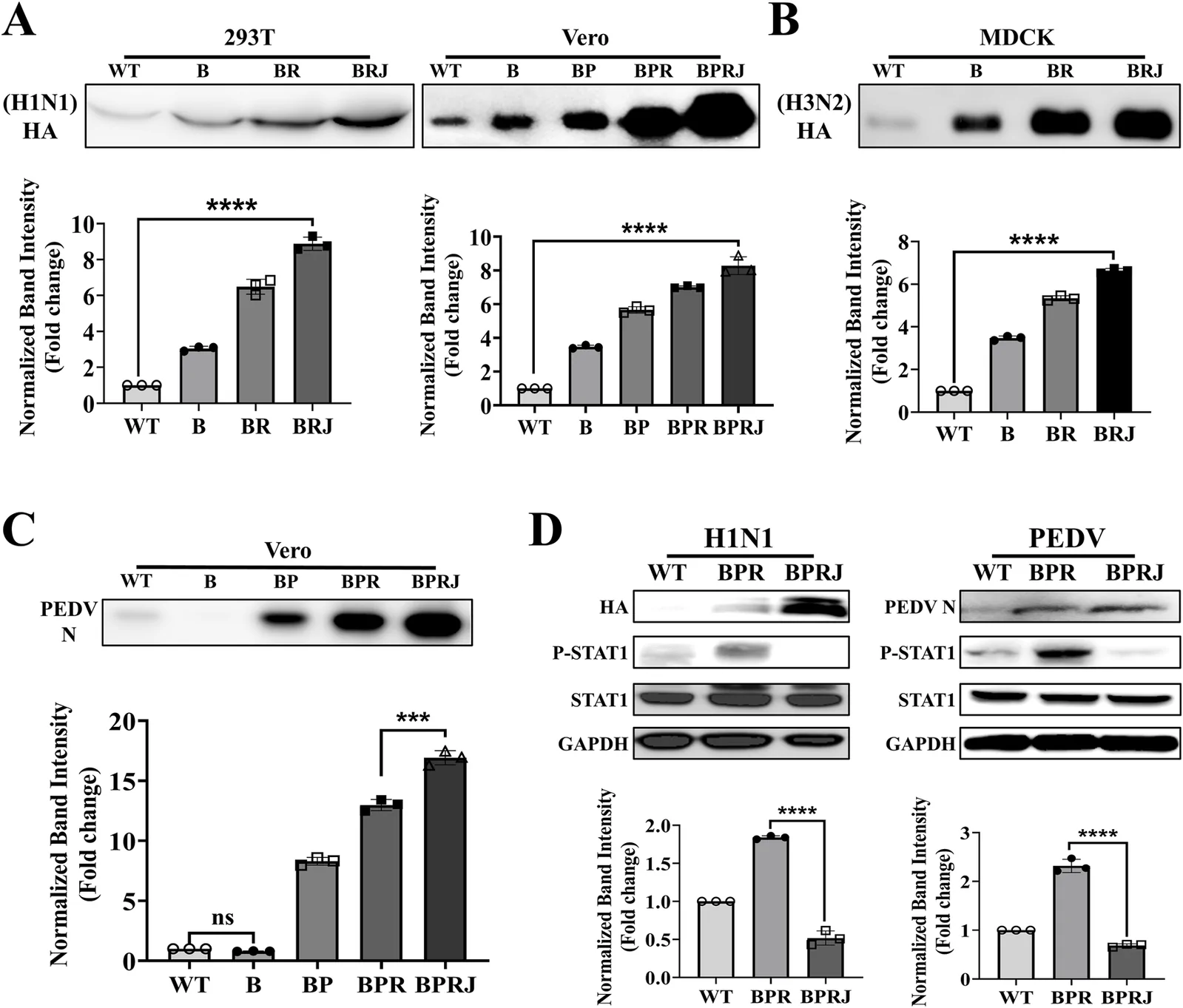
Enhanced Envelop virus production in multi-gene knockout cell lines. **(A,B)** Influenza HA expression in HEK293T, Vero, and MDCK cells increased stepwise depending on the number of knocked-out genes. Results were obtained 48 h after infection with **(A)** influenza H1N1 (A/Brisbane/59/2007) and **(B)** H3N2 (A/Brisbane/10/2007). Bottom panels: Western blot quantification results are expressed as fold change relative to wild-type cells. HA; hemagglutinin. **(C)** In Vero knockout cell lines infected with PED virus (CV777), PED N expression increased stepwise depending on the number of knocked-out genes after 48 h post-infection. **(D)** Expression of phosphorylated STAT1 (p-STAT1) 24 h after infection with influenza H1N1 virus (left panels) and PED virus (right panels). Western blot samples of panels **(A,B,D)** were prepared under reducing conditions and panel **(C)** was prepared under non-reducing conditions, while all other viral and host proteins were analyzed under reducing conditions. Statistical significance is indicated as follows: p < 0.05 (*), p < 0.01 (**), p < 0.001 (***), and p < 0.0001 (****). The results shown are representatives of at least three independent experiments.

We first assessed the platform’s ability to enhance the production of enveloped viruses, using the influenza virus as a primary model. Upon infection with influenza A/H1N1 (A/Brisbane/59/2007) or H3N2 (A/Brisbane/10/2007), hemagglutinin (HA) protein expression increased in gene knock-out number manner across 293T, Vero and MDCK engineered vaccine production cell lines. The triple or quadruple gene knockout cell lines showed increased viral output by 3 to 8-fold relative to wild-type (WT) cells, with the magnitude of enhancement being cell-specific (Figures 2A,B). This enhancement was not influenza A virus-specific, as the production of influenza B/Yamagata (B/Wisconsin/2010) in MDCK BPRJ cells also resulted in significantly higher cytopathic effect (CPE) percentage than in those of WT cells (Supplementary Figure S1B).

To evaluate the platform’s utility in veterinary vaccinology, we infected Vero background cells with the classical porcine epidemic diarrhea virus (PEDV) strain CV777. Western blot analysis of the viral nucleocapsid (N) protein demonstrated a substantial increase in viral production in the knockout cells (Figure 2C). These results collectively suggest that a targeted multi-gene knockout strategy can be applied to enhance the production of diverse enveloped viruses.

Viral infection of Vero-derived antiviral knockout cell lines demonstrated that stepwise removal of interferon-induced restriction factors markedly enhances viral production while uncoupling STAT1 activation from functional antiviral control. Following infection, BPR cells (*BST2/PKR/RNase L* triple knockout) exhibited substantially increased STAT1 phosphorylation compared with wild-type cells, reflecting heightened innate immune sensing driven by elevated viral replication. Despite this robust STAT1 activation, viral production in BPR cells was significantly increased compared to wild-type cells, indicating that the deletion of key antiviral effector proteins compromises the effective restriction of viral replication and release. In contrast, BPRJ cells (*BST2/PKR/RNase L/JAK1* quadruple knockout) displayed minimal STAT1 phosphorylation, consistent with disruption of JAK–STAT signaling and supported the highest levels of viral production among all cell lines tested (Figure 2D). These findings demonstrate that elimination of antiviral effector pathways is sufficient to increase viral yield substantially, and that additional abrogation of upstream interferon signaling further maximizes viral replication and release.

### 3.3 Enhance production of functional lentiviral vectors through the elimination of antiviral genes

**FIGURE 3:**
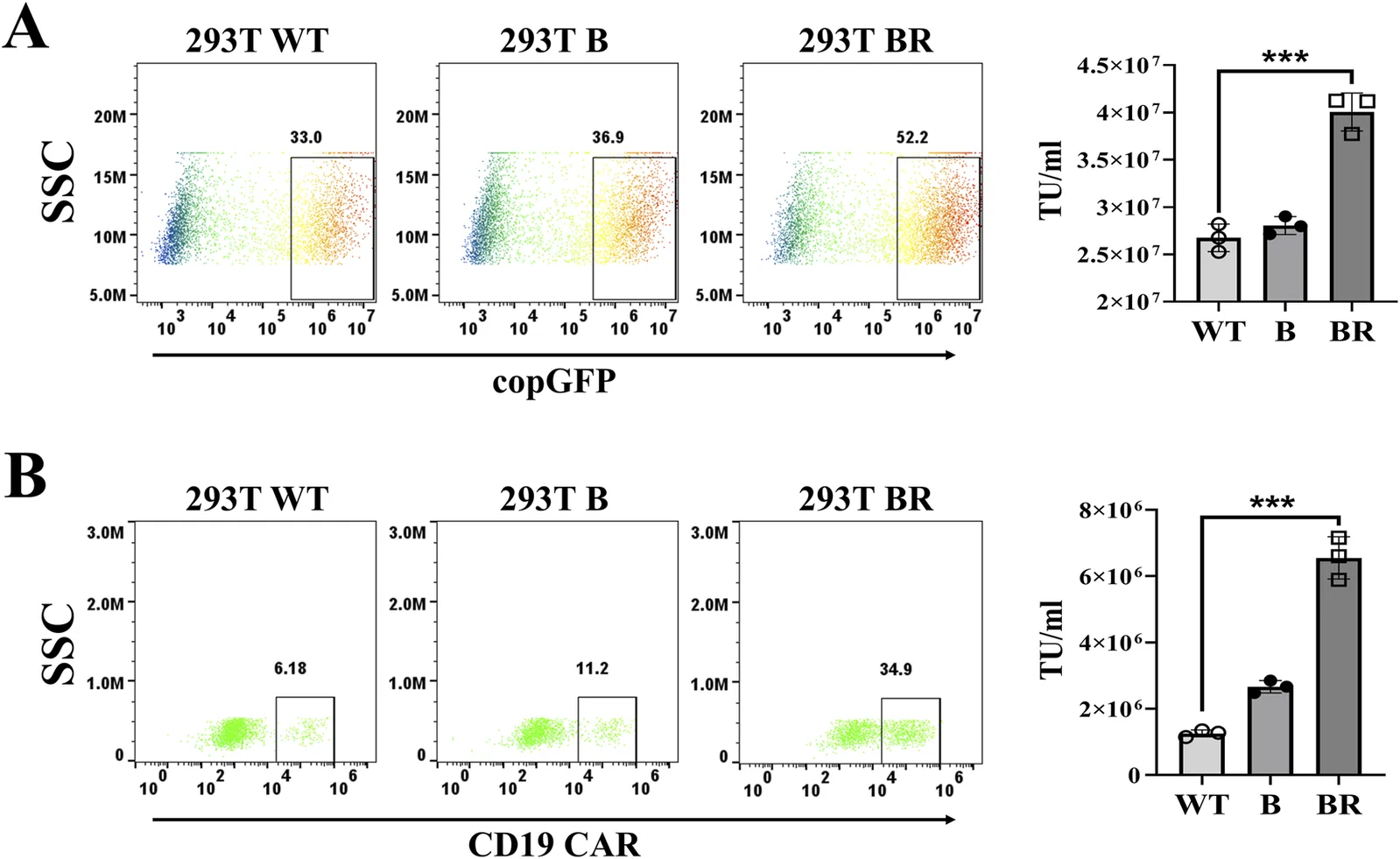
Enhanced lentivirus production in multi-gene knockout 293T cell lines. **(A)** Pseudo-typed lentiviruses carrying cop-GFP were produced in 293T WT and gene-knockout cell lines (293T B and 293T BR). Each lentivirus was transduced into 7.5 ×10^4^ of WT 293T cells at the same volume (10 μL), and the transduction units were measured. Left panels: Representative images of FACS analysis. Right panel: Transduction units calculated based on the results from left panel. **(B)** Productivity of lentiviruses containing the CD19-CAR gene in 293T WT and gene-knockout cell lines (293T B and 293T BR). Each lentivirus was transduced into 7.5 ×10^4^ of WT 293T cells at the same volume (5 μL), and the transduction units were measured. CD19-CAR was stained with protein-L-PE. Right panel: Transduction units calculated based on the results from left panel. Statistical significance is indicated as follows: p < 0.05 (*), p < 0.01 (**), p < 0.001 (***), and p < 0.0001 (****). The results shown are representative of three independent experiments.

HEK293T cells are the industry standard for producing lentiviral vectors (LVs) used in cell therapies. We produced VSV-G pseudotyped LVs carrying a copGFP reporter gene in the engineered HEK293T background cell lines. Produced viruses were transduced into HEK293T WT to evaluate the virus titer. Flow cytometry analysis of transduced HEK293T WT cells revealed that viruses produced from the BR knockout cells exhibited significantly higher functional titers (TU/mL) than those from WT cells (Figure 3A). Additionally, we evaluated the preparation of more complex LVs carrying the anti-CD19 chimeric antigen receptor (CAR). The HEK293T BR cell line consistently produced higher levels of LVs than HEK293T WT cells (Figure 3B). Functionally active CD19-CAR LV underscores the platform’s potential for advanced manufacturing of complex therapeutic vectors.

### 3.4 The enhanced viral production capacity of cell lines lacking antiviral genes extends to non-enveloped viruses as well

**FIGURE 4:**
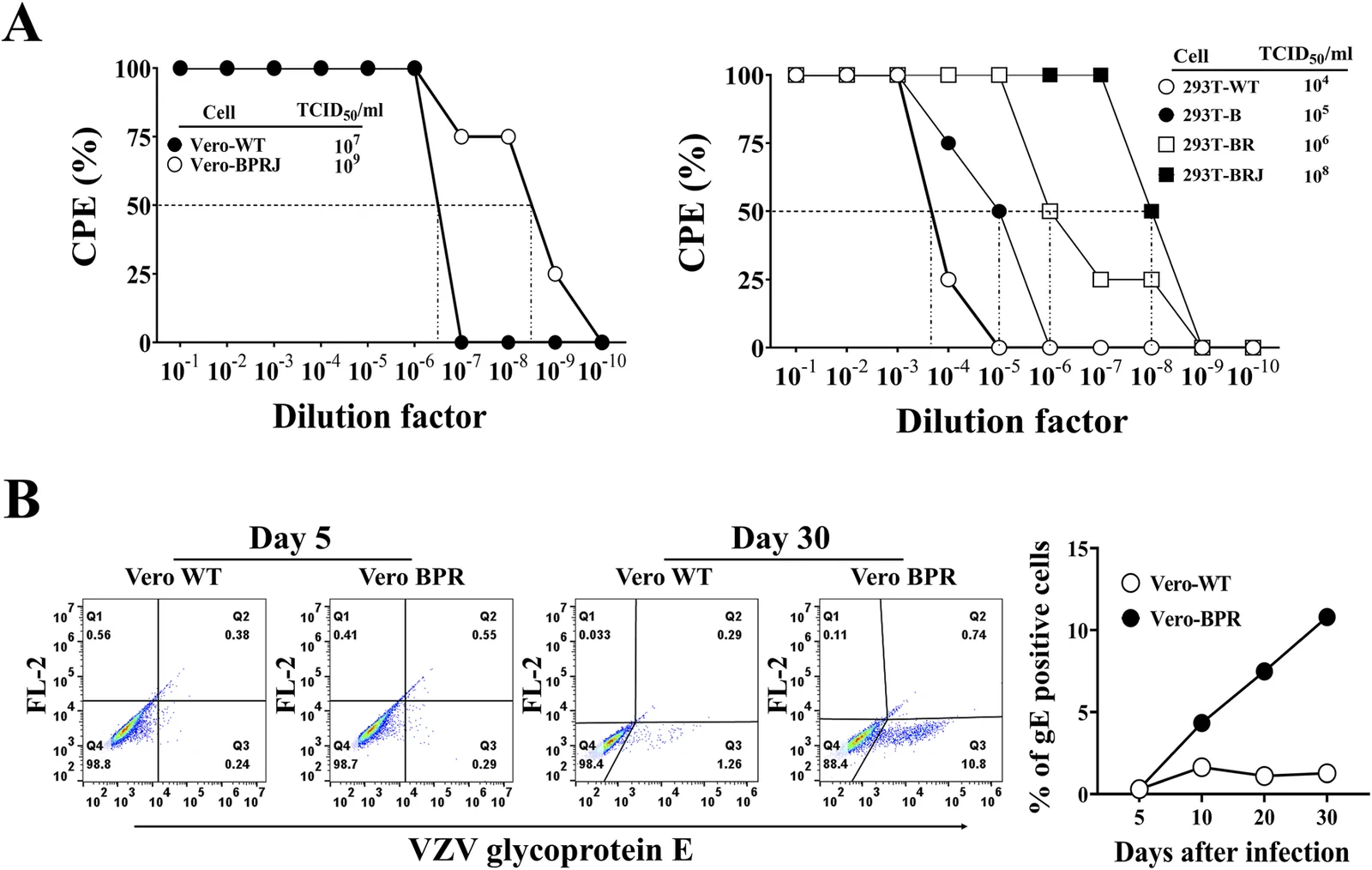
Enhanced production of non-enveloped virus and varicella zoster virus (VZV) in multi-gene knocked-out cell lines. **(A)** Production of Coxsackievirus B4 (CVB4) in gene-deficient cell lines. Left panel: Coxsackievirus B4 was produced in Vero background cell lines and their cytopathic effect (CPE) were measured in Vero WT cells. Right panel: The Coxsackievirus B3 (CVB3) replicon was transfected into 293T background cell lines, and the CPE of the produced viruses was measured in Vero WT cells. TCID50; Tissue Culture Infectious Dose 50%. **(B)** Vero WT and BPR cells were infected with VZV-Oka virus and passaged weekly. Cell surface expression of glycoprotein E was measured in both cell types during passage using flow cytometry. The results shown are representative of more than three independent experiments.

Among viruses infecting animal hosts, many possess envelopes, but non-enveloped viruses also constitute a significant proportion, and their modes of infection differ (Dimitrov, 2004; Tenge et al., 2014; Valero-Rello and Sanjuán, 2022). Having established the efficacy of our platform for enveloped viruses, we next examined whether the multi-gene knockout strategy could enhance the production of non-enveloped viruses, which BST2 does not restrict (Yi et al., 2019). Coxsackievirus B was produced in Vero WT and Vero BPRJ cells, and the harvested virus was subsequently used to infect Vero WT cells. Infection with Coxsackievirus B produced in Vero BPRJ cells resulted in a significant increase in viral titers compared to virus derived from WT cells, as determined by the CPE percentage (Figure 4A). These findings demonstrate that disrupting downstream effector pathways (PKR, RNase L) along with core signaling (JAK1) can overcome antiviral defenses of host cells against a broader range of viruses, including non-enveloped types.

Furthermore, Coxsackievirus B3 (CVB3) replicons generated in 293 T-based cells induced progressively more severe cytopathic effects (CPE) in infected Vero WT cells as the number of gene knockouts increased, indicating a positive correlation between replicon replication efficiency and the extent of host gene depletion (Figure 4A).

### 3.5 Propagation of conventionally refractory viruses in antiviral gene-deficient cell lines

Next, we tested our platform using the varicella-zoster virus (VZV). Due to its cell-associated nature and weak replication capacity, VZV is difficult to amplify in traditional cell cultures. We first infected Vero WT and Vero BPR cells with VZV-Oka virus, then passaged the infected cells to detect glycoprotein E expression at each time point. Although Vero cells were known to poorly support VZV replication, expression of VZV-Oka glycoprotein E progressively increased in passaged Vero BPR cells at each passage. In contrast, VZV-Oka glycoprotein E expression in Vero WT cells was extremely low (Figure 4B; Supplementary Figure S1C). These results demonstrate the significant potential of host cell engineering to produce difficult-to-culture human pathogens.

### 3.6 Antiviral gene-deficient cell lines adapted to suspension culture maintain enhanced viral productivity

**FIGURE 5:**
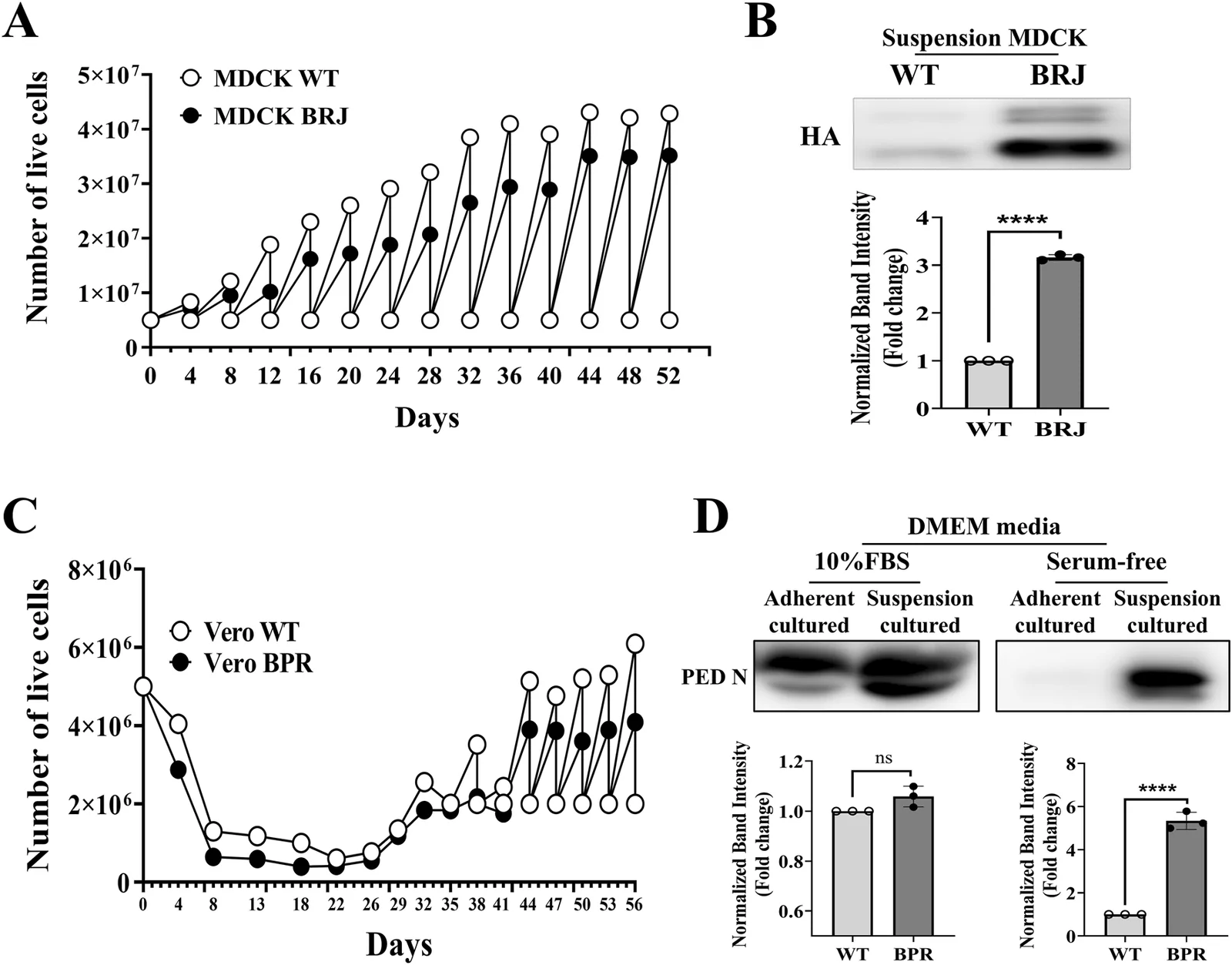
Suspension culture of multi-gene knockout cells enhances viral production. **(A)** Growth kinetics of suspension-adapted WT and MDCK BRJ cells during suspension adaptation in serum-free conditions. **(B)** HA expression levels in suspension culture were measured following H1N1 infection. 8 μL of culture supernatants were analyzed by Western blotting. **(C)** Growth kinetics of Vero WT and Vero BPR cells during suspension adaptation in serum-free conditions. **(D)** Western blot analysis of PEDV N protein in Vero BPR cells cultured in DMEM media under adherent or suspension conditions, with or without fetal bovine serum (FBS). 10 μL of culture supernatants were analyzed by Western blotting. Western blot samples of panel **(B)** were prepared under reducing conditions and panel **(D)** was prepared under non-reducing conditions.

To address the scalability limitations of adherent culture systems, we adapted the multi-gene knockout cells to suspension growth. Both MDCK WT and BRJ cells adapted in serum-free conditions until cells proliferate stably in suspension (Figure 5A). Upon H1N1 infection, HA expression in suspension-adapted MDCK BRJ cells remained significantly higher than in suspension-adapted WT cells (Figure 5B). Process optimization in shake flask cultures achieved HA yields of ≥60 μg/mL (Supplementary Figure S1D), confirming that the productivity advantage is retained in scalable formats.

Similarly, suspension-adapted Vero BPR cells infected with PEDV exhibited higher viral yields, as indicated by N protein expression, compared to that of adherent cultures. This enhancement was more pronounced under serum-free conditions (Figures 5C,D). The successful adaptation of multiple engineered cell lines to high-yield suspension culture underscores the industrial robustness and broad applicability of this synergistic platform.

## 4 Discussion

The development of robust and high-yield viral production platforms is a cornerstone of effective vaccine manufacturing and virological research (Kis et al., 2022; Joe et al., 2024). In this study, we established a versatile and highly efficient platform by systematically engineering industrially relevant cell lines-HEK293T, Vero, and MDCK (Genzel, 2015; Yang et al., 2019)-through targeted knockout of key intrinsic antiviral defense genes, including *BST2*, *JAK1*, *RNase L*, and *PKR*. This multi-target knockout approach resulted in a substantial enhancement of replication for a wide spectrum of viruses.

A central finding of our work is the consistent, knockout-gene-number-dependent enhancement of viral replication across all tested cell lines and virus families. The replication of multiple enveloped viruses, including influenza A (H1N1 and H3N2), influenza B (Yamagata), PEDV (CV777), and pseudotyped lentivirus, was significantly enhanced in the multi-gene knockout cells. This demonstrates that the redundancy inherent in the host antiviral network can be effectively overcome by a coordinated, multi-target knockout strategy, thereby surpassing the limited efficacy of single-gene edits like *BST2* (Yi et al., 2019). These findings position BST2 as a context-dependent modulator of viral replication rather than a universal restriction factor and underscore that dismantling the PKR-RNase L-JAK1 axis is pivotal for creating a broadly permissive cellular environment for viral replication (Kane et al., 2016).

Critically, the enhancement provided by our platform was not confined to enveloped viruses. The significantly increased production of Coxsackievirus B, a non-enveloped picornavirus, in Vero BPRJ cells indicates that the restrictions mediated by PKR, RNase L, and JAK-STAT signaling constitute a broad-spectrum antiviral barrier, independent of viral structure or budding mechanism. This finding significantly expands the potential utility of our platform for producing a broader range of viral biologics.

A particularly compelling result was the successful enhancement of Varicella-zoster virus (VZV) replication, a human pathogen notoriously difficult to propagate in conventional cell cultures due to its highly cell-associated nature (Laemmle et al., 2019). The progressive increase in VZV glycoprotein E expression over passages in our engineered Vero BPR-in stark contrast to the limited replication in WT controls-highlights the unique capacity of this host cell engineering strategy to enable the propagation of fastidious pathogens. This outcome holds significant promise for improving the manufacturing efficiency of live-attenuated VZV vaccines (Liu et al., 2025).

Furthermore, the marked increase in the functional titer of complex therapeutic lentiviral vectors, including a CD19-CAR construct, produced in engineered HEK293T BR and BRJ cells, underscores the direct clinical relevance of our platform. The ability to achieve higher vector titers without compromising integrity addresses a major challenge in the cost-effective and scalable manufacturing of cell therapies, such as CAR-T, thereby enhancing their clinical and commercial translation (Michels et al., 2022).

Finally, a crucial step for industrial application is the transition from a proof-of-concept in adherent culture to a scalable, industry-ready format. We demonstrated that the genetic advantages of our multi-gene knockout cells were fully retained following adaptation to suspension culture. The high yields of influenza HA protein (reaching 60 μg/mL in shake flasks) and PEDV in suspension-adapted cells confirm that our strategy successfully integrates intrinsic cellular enhancements with the bioprocess. This synergy between cellular engineering and bioprocess optimization may present a comprehensive solution to long-standing production bottlenecks (Hegde, 2015).

Although the targeting sites were carefully designed to minimize predicted off-target activity, comprehensive analyses of potential off-target mutations were not performed in this study (Yan et al., 2020). Therefore, the possibility that unintended genomic alterations contributed, at least in part, to the observed phenotype cannot be completely excluded. Future studies employing genome-wide off-target assessment will be necessary to validate further that the enhanced viral replication phenotype is specifically associated with the targeted gene knockout. Nevertheless, because the engineered cell lines described here are intended as virus-production substrates rather than products administered directly to patients, the potential impact of unintended genomic alterations is expected to be more limited than in cell-based therapeutic applications (Kalter et al., 2025).

In this study, knockout of antiviral genes consistently resulted in increased viral productivity across multiple cell lines and diverse viral species. These findings suggest that modulation of antiviral host factors may represent a broadly applicable strategy for enhancing virus production (Demirden et al., 2024). Furthermore, host-cell genetic engineering approaches, including gene knockout, may not only improve large-scale vaccine manufacturing but also facilitate the development of novel vaccines against infectious pathogens that have traditionally been difficult to propagate in cell culture (Li et al., 2013; Chen et al., 2023). Therefore, this study provides a foundation for the establishment of next-generation cell engineering platforms for vaccine development and production.

In summary, this study establishes a broadly applicable and robust framework for engineering high-efficiency viral production by engineering cell lines. By systematically targeting conserved and redundant nodes of the innate immune system, we have created a platform that significantly boosts the yield of diverse viruses, ranging from vaccine targets and therapeutic vectors to hard-to-culture pathogens. The successful adaptation of these engineered cells to suspension culture paves the way for industrial-scale manufacturing.

## Data Availability

The original contributions presented in the study are included in the article/Supplementary Material, further inquiries can be directed to the corresponding author.

## References

[B33] Au-Yeung N. Mandhana R. Horvath C. M. (2013). Transcriptional regulation by STAT1 and STAT2 in the interferon JAK-STAT pathway. Jakstat 2 (3), e23931. 10.4161/jkst.23931 24069549 PMC3772101

[B1] Chen D. Y. Turcinovic J. Feng S. Kenney D. J. Chin C. V. Choudhary M. C. (2023). Cell culture systems for isolation of SARS-CoV-2 clinical isolates and generation of recombinant virus. iScience 26 (5), 106634. 10.1016/j.isci.2023.106634 37095858 PMC10083141

[B2] Demirden S. F. Kimiz-Gebologlu I. Oncel S. S. (2024). Animal cell lines as expression platforms in viral Vaccine production: a post Covid-19 perspective. ACS Omega 9 (15), 16904–16926. 10.1021/acsomega.3c10484 38645343 PMC11025085

[B3] Dimitrov D. S. (2004). Virus entry: molecular mechanisms and biomedical applications. Nat. Rev. Microbiol. 2 (2), 109–122. 10.1038/nrmicro817 15043007 PMC7097642

[B25] Duggal N. K. Emerman M. (2012). Evolutionary conflicts between viruses and restriction factors shape immunity. Nat. Rev. Immunol. 12 (10), 687–695. 10.1038/nri3295 22976433 PMC3690816

[B35] Evans D. T. Serra-Moreno R. Singh R. K. Guatelli J. C. (2010). BST-2/tetherin: a new component of the innate immune response to enveloped viruses. Trends Microbiol 18 (9), 388–396. 10.1016/j.tim.2010.06.010 20688520 PMC2956607

[B4] Gallagher T. Lipsitch M. (2019). Postexposure effects of Vaccines on infectious diseases. Epidemiol. Rev. 41 (1), 13–27. 10.1093/epirev/mxz014 31680134 PMC7159179

[B29] García M. A. Gil J. Ventoso I. Guerra S. Domingo E. Rivas C. (2006). Impact of protein kinase PKR in cell biology: from antiviral to antiproliferative action. Microbiol. Mol. Biol. Rev. 70 (4), 1032–1060. 10.1128/mmbr.00027-06 17158706 PMC1698511

[B5] Genzel Y. (2015). Designing cell lines for viral vaccine production: where do we stand? Biotechnol. J. 10 (5), 728–740. 10.1002/biot.201400388 25903999

[B6] Hegde N. R. (2015). Cell culture-based influenza vaccines: a necessary and indispensable investment for the future. Hum. Vaccin Immunother. 11 (5), 1223–1234. 10.1080/21645515.2015.1016666 25875691 PMC4514150

[B34] Hu X. Li J. Fu M. Zhao X. Wang W. (2021). The JAK/STAT signaling pathway: from bench to clinic. Signal Transduct. Target. Ther. 6 (1), 402. 10.1038/s41392-021-00791-1 34824210 PMC8617206

[B7] Hu Y. Wu G. Jia Q. Zhang B. Sun W. Sa R. (2024). Development of a live attenuated vaccine candidate for equid alphaherpesvirus 1 control: a step towards efficient protection. Front. Immunol. 15, 1408510. 10.3389/fimmu.2024.1408510 39021566 PMC11252532

[B8] Joe C. C. D. Segireddy R. R. Oliveira C. Berg A. Li Y. Doultsinos D. (2024). Accelerated and intensified manufacturing of an adenovirus-vectored vaccine to enable rapid outbreak response. Biotechnol. Bioeng. 121 (1), 176–191. 10.1002/bit.28553 37747758 PMC10932548

[B37] Jones C. E. Tan W. S. Grey F. Hughes D. J. (2021). Discovering antiviral restriction factors and pathways using genetic screens. Gen. Virol. 102 (5), 402. 10.1099/jgv.0.001603 PMC8295917 34020727

[B9] Kalter N. Fuster-García C. Silva A. Ronco-Díaz V. Roncelli S. Turchiano G. (2025). Off-target effects in CRISPR-Cas genome editing for human therapeutics: progress and challenges. Mol. Ther. Nucleic Acids 36 (3), 102636. 10.1016/j.omtn.2025.102636 40777742 PMC12329535

[B10] Kane M. Zang T. M. Rihn S. J. Zhang F. Kueck T. Alim M. (2016). Identification of interferon-stimulated genes with antiretroviral activity. Cell Host Microbe 20 (3), 392–405. 10.1016/j.chom.2016.08.005 27631702 PMC5026698

[B11] Kis Z. Tak K. Ibrahim D. Papathanasiou M. M. Chachuat B. Shah N. (2022). Pandemic-response adenoviral vector and RNA vaccine manufacturing. NPJ Vaccines 7 (1), 29. 10.1038/s41541-022-00447-3 35236838 PMC8891260

[B12] Laemmle L. Goldstein R. S. Kinchington P. R. (2019). Modeling Varicella zoster virus persistence and reactivation - closer to resolving a perplexing persistent state. Front. Microbiol. 10, 1634. 10.3389/fmicb.2019.01634 31396173 PMC6667558

[B27] Le Tortorec A. Willey S. Neil S. J. (2011). Antiviral inhibition of enveloped virus release by tetherin/BST-2: action and counteraction. Viruses 3 (5), 520–540. 10.3390/v3050520 21994744 PMC3185764

[B13] Li X. Fan P. Jin J. Su W. An D. Xu L. (2013). Establishment of cell lines with increased susceptibility to EV71/CA16 by stable overexpression of SCARB2. Virol. J. 10, 250. 10.1186/1743-422x-10-250 23919614 PMC3765843

[B14] Liu H. Cui L. Zhang S. Wang H. Xue W. Li H. (2025). Research progress on varicella-zoster virus vaccines. Vaccines (Basel) 13 (7), 730. 10.3390/vaccines13070730 40733707 PMC12299449

[B36] Liu R. Moss B. (2016). Opposing roles of double-stranded RNA effector pathways and viral defense proteins revealed with CRISPR-Cas9 knockout cell lines and vaccinia virus mutants. J. Virol. 90 (17), 7864–7879. 10.1128/jvi.00869-16 27334583 PMC4988158

[B31] Martínez N. W. Gómez F. Tapia-Godoy A. Roa J. F. Moreso-Contreras F. Liu Y. (2025). PKR-driven ISR signaling controls synaptic translation and structural plasticity in an age-dependent manner. Neurobiol. Dis. 216, 107113. 10.1016/j.nbd.2025.107113 40976378

[B15] Michels A. Ho N. Buchholz C. J. (2022). Precision medicine: *in vivo* CAR therapy as a showcase for receptor-targeted vector platforms. Mol. Ther. 30 (7), 2401–2415. 10.1016/j.ymthe.2022.05.018 35598048 PMC9263322

[B16] Peletta A. Lemoine C. Courant T. Collin N. Borchard G. (2023). Meeting vaccine formulation challenges in an emergency setting: towards the development of accessible vaccines. Pharmacol. Res. 189, 106699. 10.1016/j.phrs.2023.106699 36796463

[B26] Randall R. E. Goodbourn S. (2008). Interferons and viruses: an interplay between induction, signalling, antiviral responses and virus countermeasures. J. Gen. Virol. 89 (Pt 1), 1–47. 10.1099/vir.0.83391-0 18089727

[B32] Sadler A. J. Latchoumanin O. Hawkes D. Mak J. Williams B. R. (2009). An antiviral response directed by PKR phosphorylation of the RNA helicase A. PLoS Pathog. 5 (2), e1000311. 10.1371/journal.ppat.1000311 19229320 PMC2637979

[B30] Silverman R. H. (2007). Viral encounters with 2',5'-oligoadenylate synthetase and RNase L during the interferon antiviral response.J. Virol. 81 (23), 12720–12729. 10.1128/jvi.01471-07 17804500 PMC2169107

[B17] Tenge V. R. Gounder A. P. Wiens M. E. Lu W. Smith J. G. (2014). Delineation of interfaces on human alpha-defensins critical for human adenovirus and human papillomavirus inhibition. PLoS Pathog. 10 (9), e1004360. 10.1371/journal.ppat.1004360 25188351 PMC4154873

[B18] Valero-Rello A. Sanjuán R. (2022). Enveloped viruses show increased propensity to cross-species transmission and zoonosis. Proc. Natl. Acad. Sci. U. S. A. 119 (50), e2215600119. 10.1073/pnas.2215600119 36472956 PMC9897429

[B28] Van Damme N. Goff D. Katsura C. Jorgenson R. L. Mitchell R. Johnson M. C. (2008). The interferon-induced protein BST-2 restricts HIV-1 release and is downregulated from the cell surface by the viral Vpu protein. Cell Host Microbe 3 (4), 245–252. 10.1016/j.chom.2008.03.001 18342597 PMC2474773

[B19] Williams B. A. Jones C. H. Welch V. True J. M. (2023). Outlook of pandemic preparedness in a post-COVID-19 world. NPJ Vaccines 8 (1), 178. 10.1038/s41541-023-00773-0 37985781 PMC10662147

[B20] Yan J. Xue D. Chuai G. Gao Y. Zhang G. Liu Q. (2020). Benchmarking and integrating genome-wide CRISPR off-target detection and prediction. Nucleic Acids Res. 48 (20), 11370–11379. 10.1093/nar/gkaa930 33137817 PMC7672467

[B21] Yang J. Guertin P. Jia G. Lv Z. Yang H. Ju D. (2019). Large-scale microcarrier culture of HEK293T cells and vero cells in single-use bioreactors. Amb. Express 9 (1), 70. 10.1186/s13568-019-0794-5 31127400 PMC6534633

[B22] Yi E. Oh J. Giao N. Q. Oh S. Park S. H. (2017). Enhanced production of enveloped viruses in BST-2-deficient cell lines. Biotechnol. Bioeng. 114 (10), 2289–2297. 10.1002/bit.26338 28498621

[B23] Yi E. Oh J. Kang H. R. Song M. J. Park S. H. (2019). BST2 inhibits infection of influenza A virus by promoting apoptosis of infected cells. Biochem. Biophys. Res. Commun. 509 (2), 414–420. 10.1016/j.bbrc.2018.12.110 30594400

[B24] Zinnecker T. Reichl U. Genzel Y. (2024). Innovations in cell culture-based influenza vaccine manufacturing - from static cultures to high cell density cultivations. Hum. Vaccin Immunother. 20 (1), 2373521. 10.1080/21645515.2024.2373521 39007904 PMC11253887

