# Supplementary material for: Systemic removal of host restriction factors enables rapid, scalable virus production for cell culture-based vaccine development
Source: Front Bioeng Biotechnol. 2026 Aug 21;14:1877497. doi: 10.3389/fbioe.2026.1877497 (PMC13541675; doi:10.3389/fbioe.2026.1877497)
Supplement: Supplementary file 1 [file Supplementaryfile1.docx]

Supplementary Material

# 1 Supplementary Figures and Tables

## 1.1 Supplementary Table 1: Cell Line-Specific Target Sequences Used in This Study

## 1.2 Supplementary Table 2: Sequence Variants Induced by Knockout

## 1.3 Supplementary Table 3: Primer Sets Used for PCR Amplification for the T7E1 Assay

Supplementary Figures

**Supplementary Figure 1. Enhanced virus production in multi-gene knockout cell lines**

(A) RNase L gene deficiency abolished IFNγ-induced RNase L expression. Arrow point to RNase L.

(B) Influenza B virus, the Yamagata (B/Wisconsin/1/2010) strain was produced in MDCK WT and BRJ cells, and then an equal volume of culture supernatant was serially diluted and inoculated into MDCK WT cells, and the virus titer was measured as the percentage of cytopathic effect.

(C) Expression of VZV glycoprotein E in Vero WT and BPR cells during serial passage of VZV (Oka strain).

(D) 100ng of recombinant HA protein (Sinobiological, 11052-V08H-50) and 4 μl of serum-free and suspension culture supernatant from MDCK-BRJ cells infected with influenza H1N1 were compared by Western blotting.
